# Comparative safety and efficacy of low- or moderate-intensity statin plus ezetimibe combination therapy and high-intensity statin monotherapy: A meta-analysis of randomized controlled studies

**DOI:** 10.1371/journal.pone.0264437

**Published:** 2022-03-04

**Authors:** Young-Mi Ah, Minseob Jeong, Hye Duck Choi

**Affiliations:** College of Pharmacy, Yeungnam University, Gyeongsan, Gyeongbuk, Republic of Korea; The University of Mississippi Medical Center, UNITED STATES

## Abstract

Statin is highly recommended for dyslipidemia to prevent atherosclerosis-related cardiovascular diseases and death. The aim of this study was to compare the efficacies and safeties of low/moderate-intensity statin plus ezetimibe combination therapy *vs*. high-intensity statin monotherapy. Meta-analysis was conducted on data included in published studies performed to compare the effects of the two treatments on lipid parameters and hs-CRP. Safety-related parameters were also evaluated. Eighteen articles were included in the meta-analysis. In terms of efficacy, low/moderate-intensity statin plus ezetimibe reduced LDL-C (SE = 0.307; 95% CI 0.153–0.463), TC (SE = 0.217; 95% CI 0.098–0.337), triglyceride (SE = 0.307; 95% CI 0.153–0.463), and hs-CRP (SE = 0.190; 95% CI 0.018–0.362) significantly more than high-intensity statin therapy. In terms of safety, the two treatments were not significantly different in terms of ALT elevation, but high-intensity statin increased AST and CK significantly more than combination therapy. This analysis indicates that low/moderate-intensity statin plus ezetimibe combined therapy is more effective and safer than high-intensity statin monotherapy, which suggests the addition of ezetimibe to statin should be preferred over increasing statin dose and that high-intensity statin should be used more carefully, especially in patients with related risks.

## Introduction

Statins (3-hydroxy-3-methylglutaryl-coenzyme A reductase inhibitors) significantly reduce the risk of cardiovascular events and are considered first-line therapies for managing dyslipidemia or atherosclerosis cardiovascular diseases (ASCVDs) [1, 2]. Several statins are currently available and are selected based on individual ASCVD risk and indicated statin intensity [3]. For instance, high-intensity statins that lower low-density lipoprotein cholesterol (LDL-C) on average by 50% or more as daily dose are strongly recommended for patients at high risk, such as those with a history of at least one major ASCVD event (e.g., recent acute coronary syndrome (ACS) or a history of myocardial infarction, ischemic stroke, or peripheral arterial disease).

Statins are generally well-tolerated, but statin-associated muscle symptoms are frequently reported and are a common cause of statin discontinuation [4]. Liver enzyme abnormalities are other notable adverse events during statin therapy [5]. Moreover, the increased risk of adverse effects on increasing dosage is a major concern. Thus, low dose statin therapy or a different treatment strategy should be considered for patients with risk factors for muscle or liver-related toxicities.

Given the increased risk posed by high-intensity statin therapy, ezetimibe is a highly recommended adjunct therapy in combination with statins [6]. When used alone ezetimibe reduces LDL-C modestly by about 18%, but greater effects can be expected for ezetimibe-statin combination therapies [6, 7]. Many prospective clinical trials have been performed to compare the efficacy and safety of high-intensity statin therapy with ezetimibe-statin (low- or moderate intensity) combination therapy. A randomized study reported that LDL-C levels were significantly lower in a group treated with atorvastatin 20 mg and ezetimibe 10 mg daily than in a group treated with atorvastatin 40 mg daily at 12 weeks after treatment commencement [8]. Liu *et al*. also suggested that statin combined with ezetimibe is more effective at reducing LDL-C than high-intensity statin monotherapy [9]. However, Oh *et al*. reported that lipid level changes achieved by rosuvastatin 20 mg *vs*. rosuvastatin 5 mg plus ezetimibe were not significantly different in patients with ACS [10].

Despite the lack of consistency of previous reports, no study systematic review or meta-analysis has been conducted on studies that compared the lipid-lowering effects of high-intensity statins versus low/moderate-intensity statin plus ezetimibe or on the safeties or adverse events of the two regimens.

The purpose of this meta-analysis was to evaluate efficacies, by determining changes in plasma lipid levels, in studies that compared low/moderate-intensity statin plus ezetimibe *vs*. high-intensity statin monotherapy. In addition, changes in safety-related parameters were evaluated and compared.

## Materials and methods

### Search strategy and study selection

We searched for published articles that compared the lipid-lowering effects and safeties of high-intensity statin (daily dose lowers LDL cholesterol on average by ≥ 50%) and low/moderate-intensity statin (daily dose lowers LDL cholesterol on average by < 50%) plus ezetimibe. Initially, we searched online databases, including MEDLINE (OVID and PubMed), EMBASE, and the Cochrane Library. The search terms used were combinations of the following PubMed MeSH terms and related text terms, that is, *statins*, *hydroxymethylglutaryl-CoA reductase inhibitors*, *3-hydroxy-3-methylglutaryl coenzyme A reductase inhibitors*, and *ezetimibe*. The bibliographies of retrieved articles and relevant reviews were also searched to identify additional eligible studies. We did not impose any publication limitations and the search was completed on 30 March 2021.

The two authors (Ah and Choi) independently reviewed and selected studies for inclusion in the systematic review. The inclusion criteria applied were as follows: (1) a randomized clinical trial; (2) the administration of high-intensity statin *vs*. low- or moderate-intensity statin plus ezetimibe; (3) inclusion of lipid concentrations; and (4) inclusion of safety data. Any disagreement regarding article inclusion was resolved by discussion. If a trial was described in more than one report, we extracted data from the most complete account and used other publications to clarify data.

The study protocol for this meta-analysis was registered in the International Prospective Register for Systematic Reviews (PROSPERO) CRD42021247742 on May 18, 2021.

### Data extraction and quality assessment

Detailed reviews of full-text articles were performed independently by the two authors. The following data were extracted from each study: first author’s surname; year of publication; country in which the work was performed; number of participants; patient characteristics; treatments given; treatment-induced changes in serum lipid concentrations and high sensitive C-reactive protein (hs-CRP); and adverse events. The methodological quality of each trial was evaluated by two authors using the Jadad scale [11]. This scale evaluates randomized controlled trials using the following five indicators: an adequate description of how randomization was achieved; appropriateness of the randomization method; an adequate account of how the investigators were double-blinded; appropriateness of the double-blinding method chosen; and details on patient withdrawal and dropout. A score of greater than three was considered to reflect high-quality work. Any disagreement between the two authors was resolved by discussion.

### Meta-analysis of efficacy and safety

The efficacy endpoints used were changes in lipid concentrations, including changes in LDL-C, HDL-C, total cholesterol (TC), triglycerides, apolipoprotein (Apo) A1, and Apo B. Changes in hs-CRP were also analyzed. Standard difference in means (SE) with a 95% confidence interval (CI) wererespectivley calculated to assess the effects of high-intensity statin monotherapy and low/moderate-intensity statin plus ezetimibe.

To evlauate treatment safeties, we measured differences between treatment-induced elevations of alanine aminotransferase (ALT), aspartate aminotransferase (AST), and creatine phosphokinase (CK). Likewise, mean changes, using CI of 95% CI, were calculated to assess the influences of the two treatments.

### Statistical analysis

Study heterogeneity was assessed using the χ^2^ test (employing Q statistics) and quantified by calculating *I*^2^ values [12]. A fixed-effects model (the Mantel-Haenszel method) or a random-effects model (the DerSimonian-Laird method) was applied based on the results of heterogeneity testing [13, 14].

Sensitivity analyses were performed by excluding the contributions of each study to the meta-analysis data in turn. Potential publication bias was examined using Begg’s and Egger’s tests [15, 16].

The analysis was performed using Comprehensive Meta-analysis Software version 2 (CMA 26526; Biostat, Englewood, NJ). All statistical tests were two-sided and statistical significance was accepted for P values <0.05.

## Results

### Study qualities and characteristics

**Fig 1:**
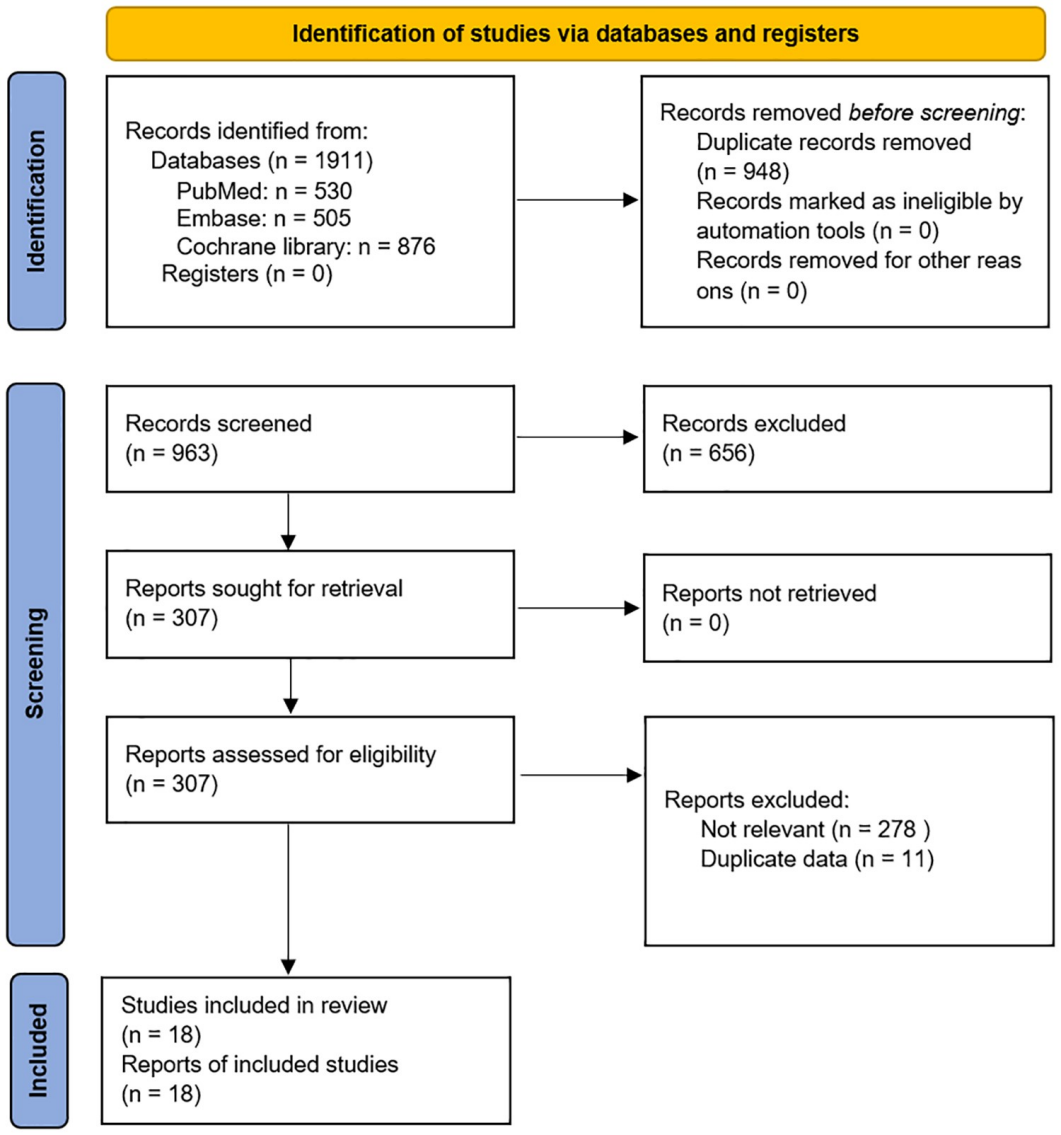
PRISMA diagram of the process for selection of relevant studies.

In total, 1,911 articles were identified during the literature search. After removing duplicates, the titles and abstracts of 963 articles were screened. Of these, 656 articles were excluded, and the full texts of the remaining 307 articles were assessed in terms of eligibility. A further 289 articles were then excluded, and data from the remaining 18 articles were included in the meta-analysis. Fig 1 shows the study selection flow chart according to PRISMA 2020 flow diagram [17]. Using the Jadad system, 9 studies were classified as low quality (scores of ≤ 2) and the other nine studies as high quality (scores of ≥ 3) (Table 1).

**Table 1:** General characteristics of the included studies for meta-analysis.

Study	Design	Participants	Duration	Intervention	No	Age, year^a^	Male, %	Diabetes, %	HTN, %	Smoker, %	Jadad score
Oh, 2020 [10]	Open-label, parallel	ACS	6 months	R20 R5 + E10	25 25	59.2 ± 9.7 59.6 ± 9.9	92.0 84.0	20.2 16.7	28.0 45.8	36.0 37.5	3
Liu, 2018 [9]	Parallel	CHD	8 weeks	A30 A20 + E10	60 60	60 ± 8.5 60 ± 8.7	61.7 66.7	NA	58.3 55.0	46.7 48.3	2
Wu, 2018 [8]	Open-label, parallel	ASCVD	12 weeks	A40 A20 + E10	50 48	57 ± 8 56 ± 11	72.0 72.9	NA	NA	NA	2
Ran, 2017 [18]	Open-label, parallel	Non-STE ACS	12 weeks	R20 R10 + E10	41 42	60.5 ± 10.0 60.4 ± 8.2	73.2 76.2	26.8 26.2	48.8 50.0	53.7 54.8	3
Yang, 2017 [19]	Double-blind, placebo-controlled	High cardiovascular risk	12 weeks	R20 R5 + E10 R10 + E10	41 40 41	62.7 ± 9.6 64. 8 ± 8.2 62.1 ± 9.5	66.7 55.3 65.0	56.4 36.8 42.5	61.9 71.1 60.5	17.9 18.4 10.0	3
Japaridze, 2016 [20]	Open-label, parallel	ACS	16 weeks	A40-80 A20-40 + E10	146 146	62.62 ± 11.03 62.21 ± 11.36	53.1 54.1	1.4 4.8	NA	NA	2
Pytel, 2016 (1) [21]	Parallel	CAD	6 months	R20 A40 A10 + E10	21 20 20	62 ± 71	NA	NA	NA	NA	2
Pytel, 2016 (2) [22]	Parallel	CAD	6 months	A40 A10 + E10 R 15	12 6 10	63 ± 7	NA	NA	NA	NA	2
Villegas-Rivera, 2015 [23]	Double-blind, parallel	Type 2 diabetes	16 weeks	R20 S20 + E10	25 25	54.0 ± 10.5 55.0 ± 12.0	48 40	100 100	NA	48 32	5
Deharo, 2014 [24]	Open-label, parallel	ACS	1 months	R20 S40 + E10	64 64	59.4 ± 11.22 58.4 ± 10.9	91 86	16 26	NA	45 55	2
Moreira, 2014 [25]	Open-label, parallel	Dyslipidemia	12 weeks	R80 S40 + E10	57 55	NA	NA	NA	NA	NA	2
Westerink, 2013 [26]	Double-blind, cross-over	Obesity with metabolic syndrome	6 weeks	S80 S10 + E10	93	57 ± 9	59	NA	NA	NA	3
Araujo, 2010 [27]	Cross-over	Hypercholesterolemia	4 weeks	S80 S10 + E10	23	NA	NA	NA	NA	NA	2
Hajer, 2009 [28]	Double-blind, cross-over	Obesity with metabolic syndrome	6 weeks	S80 S10 + E10	19	54 ± 7	100	NA	NA	NA	3
Ostad, 2009 [29]	Double-blind, parallel	CAD	8 weeks	A80 A10 + E10	24 25	66 ± 9 64 ± 10	79 76	25 16	88 68	17 32	3
Olijhoek, 2008 [30]	Double-blind, cross-over	Metabolic syndrome	6 weeks	S80 S10 + E10	19	54 ± 7	NA	NA	NA	NA	3
Settergren, 2008 [31]	Double-blind, parallel	Type 2 diabetes or IGT and CAD	6 weeks	S80 S10 + E10	20 19	70 74	75 58	85 100	NA	20 21	5
Piorkowski, 2007 [32]	Parallel	CAD	4 weeks	A40 A10 + E10	25 26	61.4 ± 1.8 62.0 ± 2.1	76.9 88.0	15.4 28.0	NA	69.2 64.0	2

Abbreviations: A, atorvastatin; ACS, acute coronary syndrome; ASCVD, atherosclerotic cardiovascular disease; CAD, carotid artery disease; CHD, coronary heart diseases; E, ezetimibe; IGT, impaired glucose tolerance; NA, not reported; Non-STE, non-ST segment elevation; R, rosuvastatin; S, simvastatin.

^a^ Values are presented means or means ± standard deviations.

### Meta-analysis of lipid parameters and hs-CRP

**Fig 2:**
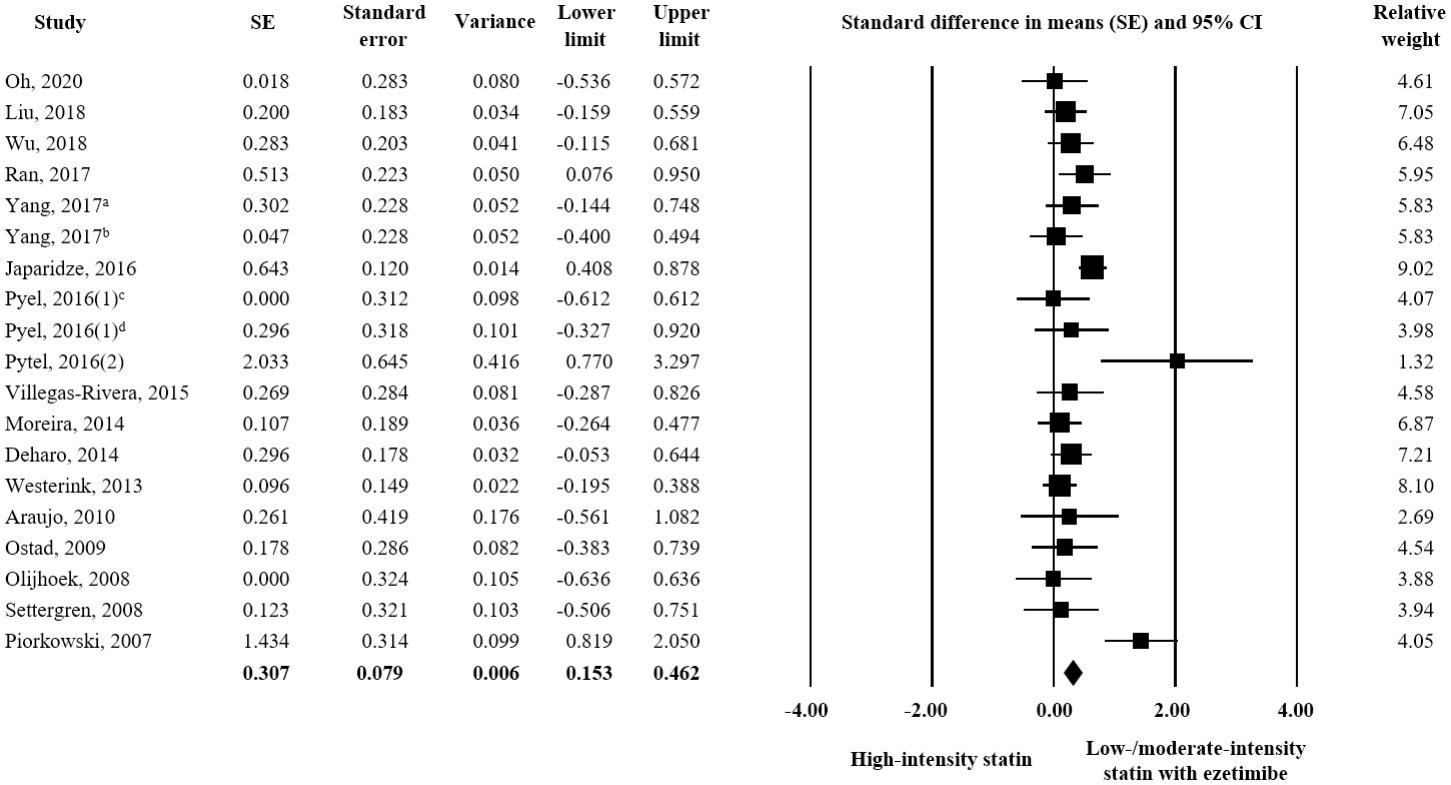
Forest plot of efficacy evaluation results. Changes in LDL-C observed for high-intensity statin monotherapy and low/moderate-intensity statin plus ezetimibe combination therapy. ^a^ rosuvastatin 20 mg vs. rosuvastatin 5 mg plus ezetimibe 10 mg; ^b^ rosuvastatin 20 mg vs. rosuvastatin 10 mg plus ezetimibe 10 mg; ^c^ rosuvastatin 20 mg vs. atorvastatin 10 mg plus ezetimibe 10 mg; ^d^ atorvastatin 40 mg vs. atorvastatin 10 mg plus ezetimibe 10 mg.

Seventeen studies measured changes in LDL-C in 787 participants treated with high-intensity statin and 752 participants treated with low/moderate-intensity statin plus ezetimibe. Combination treatment afforded a significantly greater reduction in LDL-C than high-intensity statin treatment (SE = 0.307; 95% CI 0.153–0.462) (Fig 2). Additionally, 9 studies with high-quality were included for subgroup analysis to consider the result of quality assessment, but the same result was obtained as the main analysis (data available upon request).

**Fig 3:**
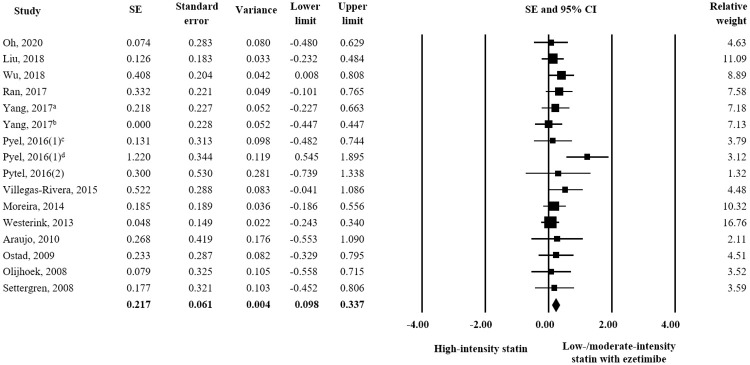
Forest plot of efficacy evaluation results. Changes in TC observed for high-intensity statin monotherapy and low/moderate-intensity statin plus ezetimibe combination therapy. ^a^ rosuvastatin 20 mg vs. rosuvastatin 5 mg plus ezetimibe 10 mg; ^b^ rosuvastatin 20 mg vs. rosuvastatin 10 mg plus ezetimibe 10 mg; ^c^ rosuvastatin 20 mg vs. atorvastatin 10 mg plus ezetimibe 10 mg; ^d^ atorvastatin 40 mg vs. atorvastatin 10 mg plus ezetimibe 10 mg.

**Fig 4:**
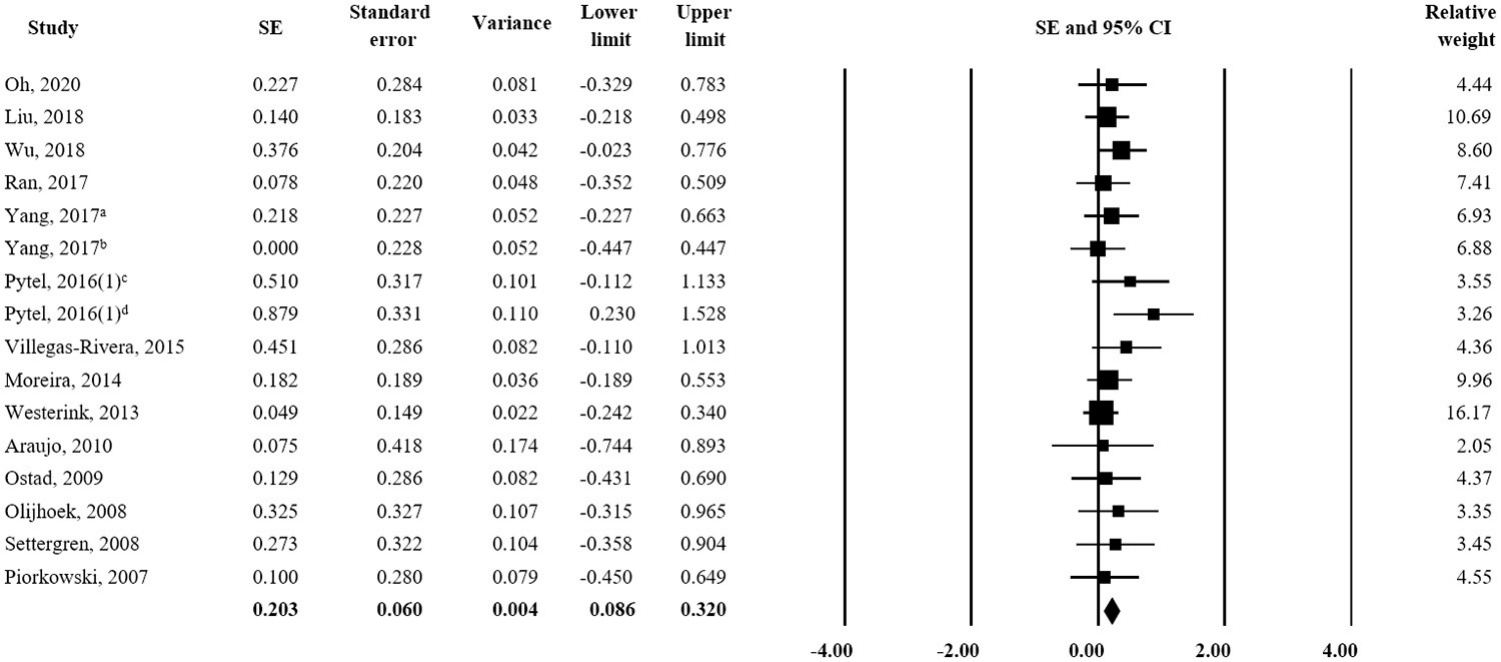
Forest plot of efficacy evaluation results. Changes in Triglyceride observed for high-intensity statin monotherapy and low/moderate-intensity statin plus ezetimibe combination therapy. ^a^ rosuvastatin 20 mg vs. rosuvastatin 5 mg plus ezetimibe 10 mg; ^b^ rosuvastatin 20 mg vs. rosuvastatin 10 mg plus ezetimibe 10 mg; ^c^ rosuvastatin 20 mg vs. atorvastatin 10 mg plus ezetimibe 10 mg; ^d^ atorvastatin 40 mg vs. atorvastatin 10 mg plus ezetimibe 10 mg.

**Fig 5:**
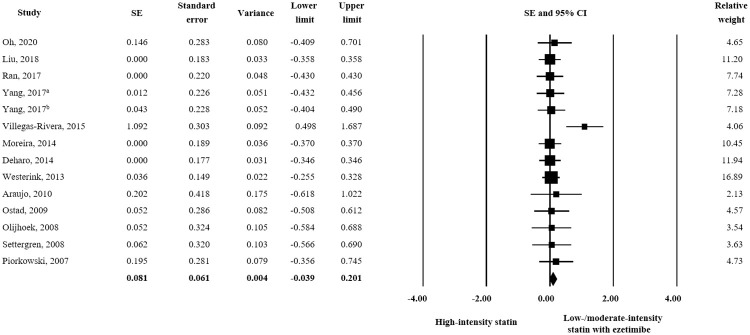
Forest plot of efficacy evaluation results. Changes in HDL-C observed for high-intensity statin monotherapy and low/moderate-intensity statin plus ezetimibe combination therapy. ^a^ rosuvastatin 20 mg vs. rosuvastatin 5 mg plus ezetimibe 10 mg; ^b^ rosuvastatin 20 mg vs. rosuvastatin 10 mg plus ezetimibe 10 mg.

**Fig 6:**
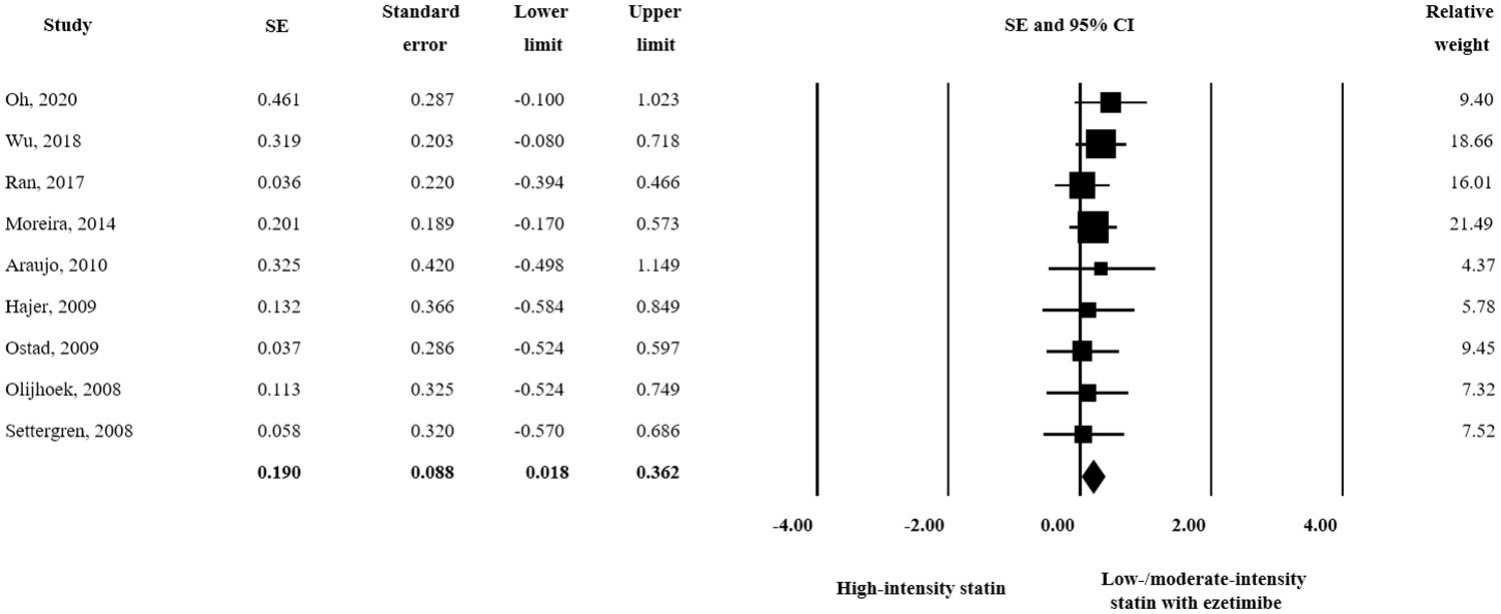
Forest plot of efficacy evaluation results. Changes in hs-CRP observed for high-intensity statin monotherapy and low/moderate-intensity statin plus ezetimibe combination therapy.

Fourteen studies assessed changes in TC and triglyceride and low/moderate-intensity statin plus ezetimibe produced significantly better responses than high-intensity statin alone (TC: SE = 0.217; 95% CI 0.098–0.337, triglyceride: SE = 0.203; 95% CI 0.086–0.320) (Figs 3 and 4). On the other hand, an analysis of 13 studies revealed no significant difference for the effects of treatments on HDL-C (Fig 5) or ApoA1 or ApoB (HDL-C: SE = 0.081; 95% CI -0.039–0.201, ApoA1: SE = 0.087; 95% CI -0.113–0.287, and ApoB: SE = 0.132; 95% CI -0.067–0.331).

hs-CRP changes were assessed in 9 studies, and low/moderate-intensity statin plus ezetimibe had a significantly greater effect than high-intensity statin therapy (SE = 0.190; 95% CI 0.018–0.362) (Fig 6).

### Meta-analysis of adverse events

**Fig 7:**
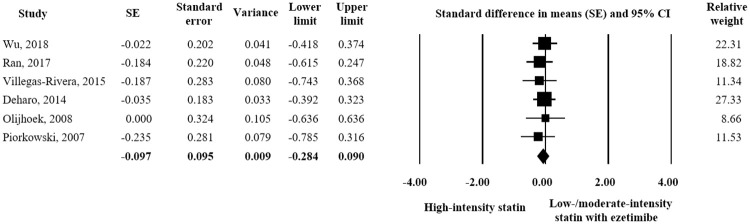
Forest plot of safety evaluation results. Changes in ALT observed for high-intensity statin monotherapy and low/moderate-intensity statin plus ezetimibe combination therapy.

**Fig 8:**
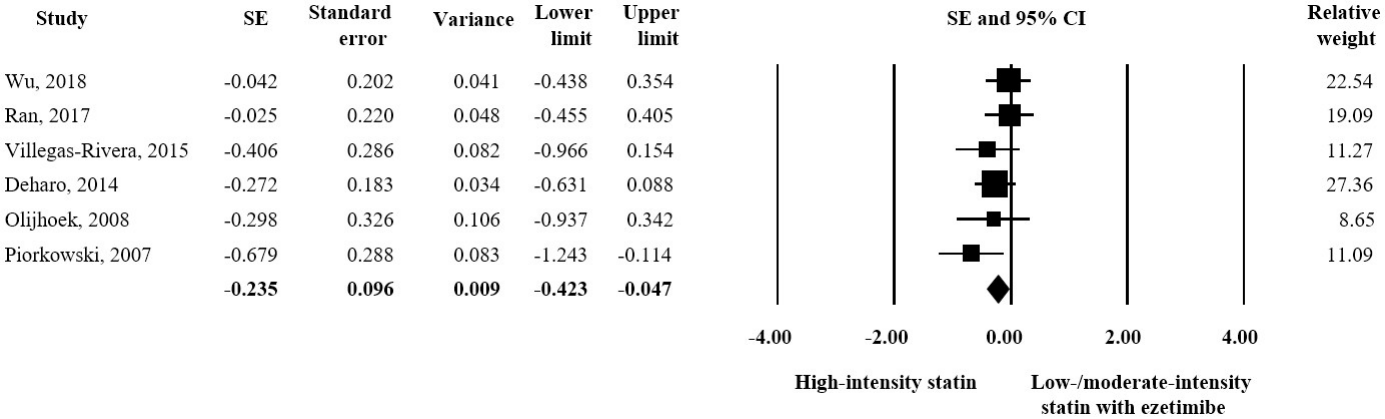
Forest plot of safety evaluation results. Changes in AST observed for high-intensity statin monotherapy and low/moderate-intensity statin plus ezetimibe combination therapy.

**Fig 9:**
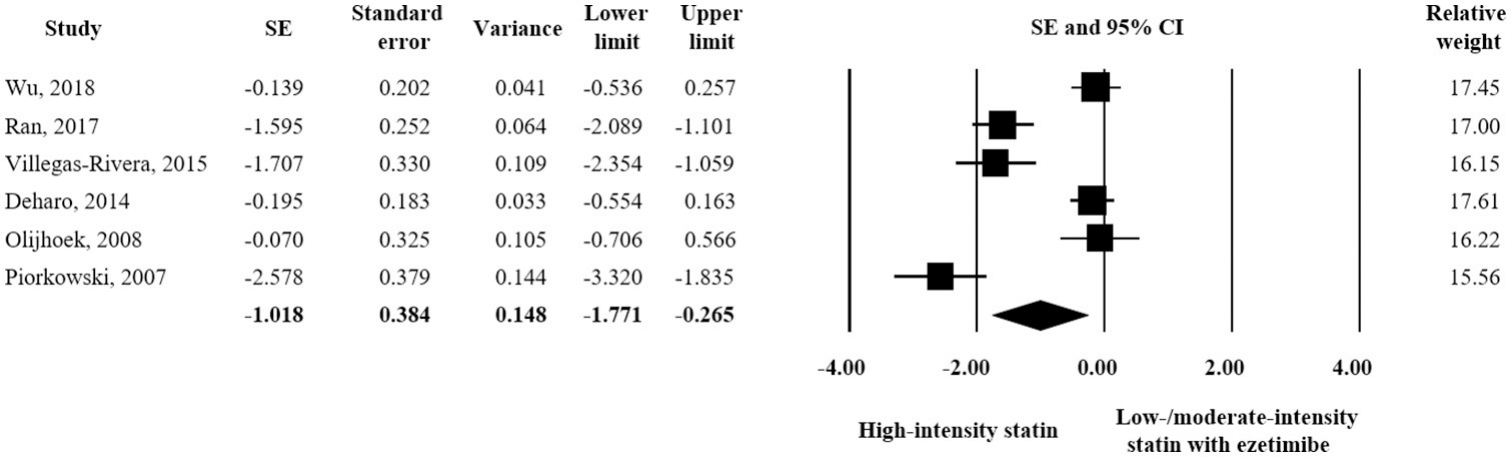
Forest plot of safety evaluation results. Changes in CK observed for high-intensity statin monotherapy and low/moderate-intensity statin plus ezetimibe combination therapy.

Six studies and 440 participants (220 for each treatment) were included in the adverse event assessment. As regards liver-related toxicity, no significant difference was observed between the two treatments in terms of ALT elevation. (SE = 0.-097; 95% CI -0.284–0.090) (Fig 7). However, high-intensity statin was associated with a significantly greater AST level than combination treatment (SE = -0.235; 95% CI -0.423–-0.047) (Fig 8). In terms of muscle-related toxicity, high-intensity statin resulted in a significantly greater increase in CK than low-intensity statin plus ezetimibe (SE = -1.018; 95% CI -1.771–-0.265) (Fig 9).

### Sensitivity analysis and publication bias

Sensitivity analysis was performed by recalculating all findings after omitting data from individual studies. With the exception of ApoA1, findings were not altered significantly (data available on request). We also evaluated publication bias; the results of Begg’s rank-correlation test and Egger’s regression test are shown in Table 2.

**Table 2:** Test of heterogeneity and publication bias.

No. of study		Test of heterogeneity			Publication bias	
		*Q* value	*P* value	*I* ^2^	*P* value (Begg’s)	*P* value (Egger’s)
*Efficacy*						
LDL-C	17	36.94	0.005	51.26	0.221	0.443
TC	14	13.80	0.541	0.000	0.047	0.075
Triglyceride	14	9.389	0.856	0.000	0.014	0.026
HDL-C	13	12.39	0.496	0.000	0.001	0.062
Apo A1	5	0.657	0.957	0.000	0.231	0.094
Apo B	5	6.916	0.140	42.16	0.043	0.098
Hs-CRP	9	2.436	0.965	0.000	0.301	0.432
*Safety*						
ALT	6	0.842	0.974	0.000	0.354	0.211
AST	6	4.636	0.462	0.000	0.226	0.121
CK	6	65.46	0.000	92.36	0.030	0.057

Abbreviations: LDL-C, low-density lipoprotein cholesterol; TC, total cholesterol; HDL-C, high-density lipoprotein cholesterol; Apo, apolipoprotein; hs-CRP, higsensitive C-reactive protein; ALT, alanine aminotransferase (ALT); AST, aspartate aminotransferase; CK. creatine phosphokinase.

## Discussion

We performed this meta-analysis to compare the safety and efficacy of high-intensity statin monotherapy and low/moderate-intensity statin plus ezetimibe combination therapy. We found that combination therapy better improved LDL-C, TC, and triglyceride than monotherapy. AST and CK levels were increased significantly more by high-intensity statin therapy.

Based on a meta-analysis of lipid parameters, we suggest that adding ezetimibe to statin therapy should be preferred to increasing statin dose, because LDL-C is known to be closely associated with ASCVDs or mortality and is of great clinical importance for the management of dyslipidemia [33, 34]. Furthermore, combination therapy had greater beneficial effects on TC and triglyceride than statin monotherapy, which we consider a notable outcome. However, both treatments had minimal effects on HDL-C.

The role of non-HDL-C in lipid management to prevent ASCVD has been reported and become clearer in recent years. It can be a better indicator than LDL-C if patients have hypertriglyceridemia, diabetes, metabolic syndrome, or chronic kidney disease [35, 36]. The value of Non-HDL-C is simply calculated as TC minus HDL-C, so the reduction in TC leads to lower non-HDL-C. Triglycerides are also assocated with the risk of ASCVD, but thier dierct role in the development of ASCVD remains controversial [37]. Our meta-analysis has clinical significance in that we found the differences in these lipid levels between the two therapies.

hs-CRP is a predictor of cardiovascular risk, as evidenced consistently by reports on the topic [38–40]. When we evaluated the effects of the two treatments on hs-CRP, combination treatment was found to have the greater effect, and in a previous meta-analysis, a significant positive relationship was found between changes in CRP, a marker of inflammation in atherosclerosis, and changes in LDL-C and [41], which is in-line with our observations.

Regarding the safety analysis, elevations in AST and CK were significantly higher in patients treated with high-intensity statin monotherapy. Treatment-related ALT elevations were not significantly different for the two treatments and reported adverse events were usually tolerable. Ezetimibe is known to be safe and well-tolerated, though mild gastrointestinal adverse events have been reported [42]. ALT elevations and myalgia have also been reported for ezetimibe/statin combinations, but reported adverse events were putatively attributed to the use of statins at high dosages [43]. Thus, our safety analysis cautions that high-intensity statin therapy requires careful consideration in patients with related risk factors, and that low/moderate-intensity statin plus ezetimibe combination therapy is a more rational choice based on considerations of efficacy and safety.

A similiar meta-analysis comparing the effects of high-dose statins or low-dose statins in combination with ezetimibe on endothelial function has been previously reported [44]. Detection of endothelial dysfunction before a clinically important plaque burden manifests is a meaningful approach as it may help to identify some patients at higher risk of future cardiovascular events [45]. However, the effects on endothelial function was not significantly different between the two treatments and there was no difference in LDL-C changes, which is inconsistent with our meta-analysis. Consequently, the findings from previous meta-analysis have been updated through our meta-analysis that include a larger number of recent studies.

In the results of heteroginiety test, the *I*^*2*^ vlaues of LDL-C and Apo1 were 51.26 and 42.16, respectively, indicating that there was an intermediate level of heteroginiety (Table 2). Therefore, reanalysis was performed using a random-effects model and the results for statistical significance were the same. That is, it suggests that some heteroginiety exists, but the influence on meta-analysis is insignificant.

There were limitations to our study as below. The present meta-analysis was performed using data abstracted from previous reports, which were not necessarily complete or accurate. In addition, the results could be partially different from the evaluations of safety or efficacy when applied to individual patients. Despite limitations, we believe our meta-analysis is meaningful because it provides clinical evidence suggesting low/moderate-intensity statin plus ezetimibe provides a better pharmacotherapeutic option than high-intensity statin monotherapy in patients with dyslipidemia.

## Conclusions

This meta-analysis shows that low/moderate-intensity statin plus ezetimibe improved lipid levels more than high-intensity statin monotherapy, and that statin monotherapy increased ALT and CK more than statin/ezetimibe combination therapy. Therefore, we recommend low/moderate-intensity statin plus ezetimibe therapy be adopted rather than increasing statin dose, and that increasing statin dose should be applied judiciously, especially in patients with related risks.

## Supporting information

S1 Fig Funnel plot for LDL-C. (TIF)

S1 Checklist (DOCX)
