# Supplementary figures and images for: Comparative safety and efficacy of low- or moderate-intensity statin plus ezetimibe combination therapy and high-intensity statin monotherapy: A meta-analysis of randomized controlled studies
Source: PLoS One. 2022 Mar 4;17(3):e0264437. doi: 10.1371/journal.pone.0264437 (PMC8896700; doi:10.1371/journal.pone.0264437)

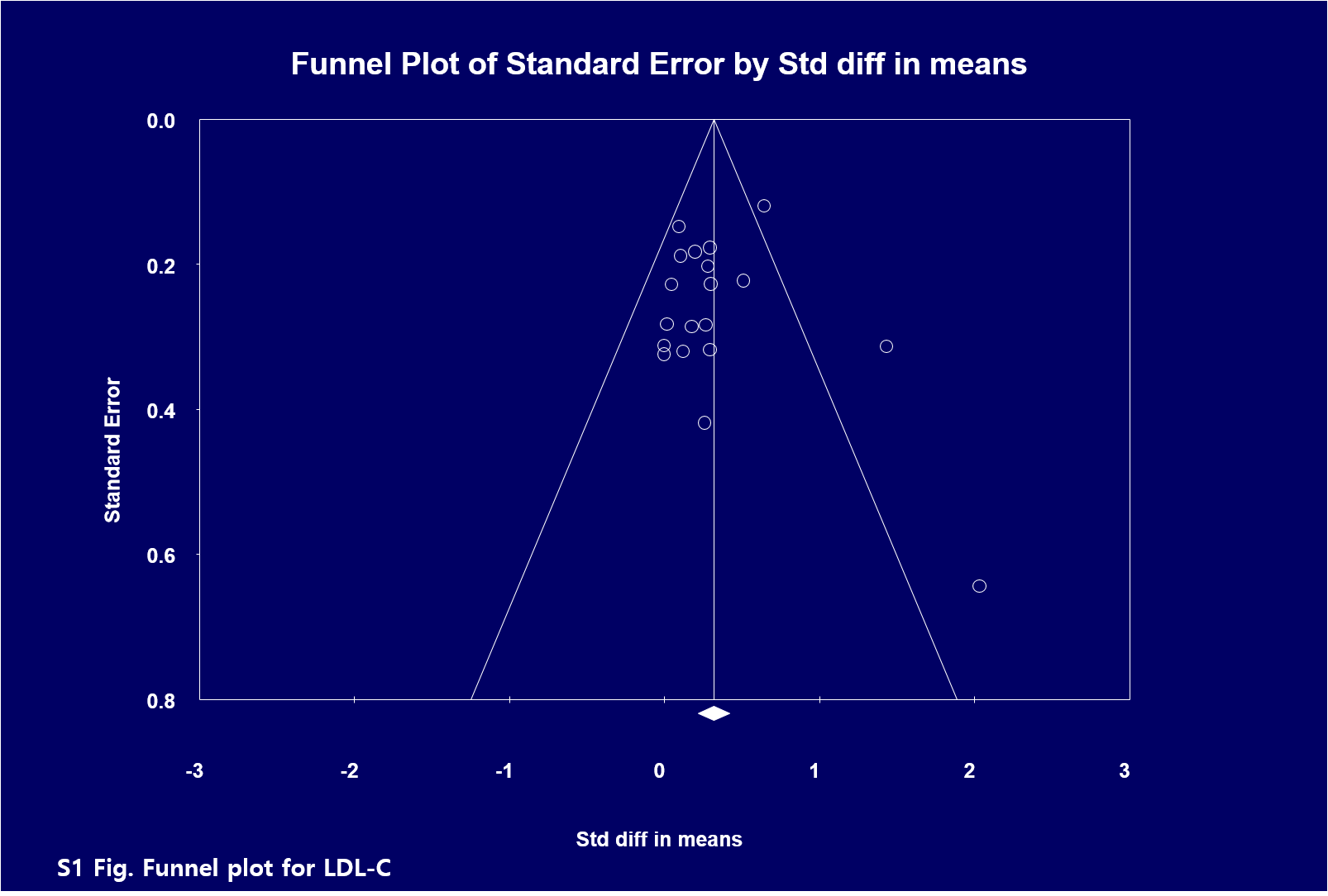

Supplement: S1 Fig — (TIF) [file pone.0264437.s001.tif]
